# Revealing the difference of *α-amylase* and *CYP6AE76* gene between polyphagous *Conogethes punctiferalis* and oligophagous *C. pinicolalis* by multiple-omics and molecular biological technique

**DOI:** 10.1186/s12864-022-08753-9

**Published:** 2022-07-19

**Authors:** Dapeng Jing, Sivaprasath Prabu, Tiantao Zhang, Shuxiong Bai, Kanglai He, Yongjun Zhang, Zhenying Wang

**Affiliations:** State Key Laboratory for Biology of Plant Diseases and Insect Pests, Institute of Plant Protection, Chinese Academy of Agricultural Sciences, Beijing, 100193 China

**Keywords:** *Conogethes punctiferalis*, *Conogethes pinicolalis*, Transcriptomics, Proteomics, Metabolomics, Gene mutation

## Abstract

**Background:**

*Conogethes pinicolalis* has been thought as a Pinaceae-feeding variant of the yellow peach moth, *Conogethes punctiferalis*. The divergence of *C. pinicolalis* from the fruit-feeding moth *C. punctiferalis* has been reported in terms of morphology, ecology, and genetics, however there is a lack of detailed molecular data. Therefore, in this study, we investigated the divergence of *C. pinicolalis* from *C. punctiferalis* from the aspects of transcriptomics, proteomics, metabolomics and bioinformatics.

**Results:**

The expression of 74,611 mRNA in transcriptome, 142 proteins in proteome and 218 metabolites in metabolome presented significantly differences between the two species, while the KEGG results showed the data were mainly closely related to metabolism and redox. Moreover, based on integrating system-omics data, we found that the *α-amylase* and *CYP6AE76* genes were mutated between the two species. Mutations in the *α-amylase* and *CYP6AE76* genes may influence the efficiency of enzyme preference for a certain substrate, resulting in differences in metabolic or detoxifying ability in both species. The qPCR and enzyme activity test also confirmed the relevant gene expression.

**Conclusions:**

These findings of two related species and integrated networks provide beneficial information for further exploring the divergence in specific genes, metabolism, and redox mechanism. Most importantly, it will give novel insight on species adaptation to various diets, such as from monophagous to polyphagous.

**Supplementary Information:**

The online version contains supplementary material available at 10.1186/s12864-022-08753-9.

## Background

*Conogethes punctiferalis* (Guenée), is an important agricultural pest of chestnut (*Castanea mollissima*), peach (*Amygdalus persica*), apple (*Malus pumila*), maize (*Zea mays*), and sunflower (*Helianthus annuus*) [1]. In some regions of China, it has become the main pest on corn, causing more significant damages than *Ostrinia furnacalis* (Guenée), the most prevalent corn pest in China [2]. *C. pinicolalis* (Lepidoptera: Crambidae) is a sibling species of *C. punctiferalis*, even though they were considered the same species at the early stage. Koizumi first identified and classified the *C. pinicolalis* as another type of *C. punctiferalis* commonly known Pinaceae-feeding type in 1963 [3]. Honda and Mitsuhashi distinguished the differences in the adults, larvae and pupal stages between the two [4]. Konno et al. reported that they were different species from their responses to different spectra of host plant constituents [5]. Finally, the pinaceae-feeding type was named as *C. pinicolalis* in 2006 [6].

These two sibling species, *C. punctiferalis* and *C. pinicolalis,* are important pest species in China. They are quite similar on morphology, almost indistinguishable between egg, larva and pupa, but only a little differences in adults. Moreover, they are also similar in response to (E)-10-hexadecenal (E10–16:Ald) and (Z)-10-hexadecenal (Z10–16:Ald) which is their main sex pheromone components, although their foraging ranges are widely differentiated. *C. punctiferalis* is a polyphagous species posing a major threat to over 100 essential plant species [7], while *C. pinicolalis* is an oligophagous insect, mainly feeding on pine trees, especially Masson pine (*Pinus massoniana*). Their feeding preferences may be associated with olfactory and gustatory system or digestive system, and this characteristic unexpectedly resulted from the expressions of genes, proteins and pathways. Therefore, it was of great interest to unveil the differences in some functional genes or proteins between the two species.

In this study, we applied a proteomic technique, the isobaric tags for relative and absolute quantification (iTRAQ), and RNA sequencing-based transcriptome technique. The transcripts with new exons were identified from an alternative splicing database to understand further the related proteins and transcripts involved in feeding preferences. Metabolomics was also used to detect the differences between the two species. Our result aims to provide a profound understanding of the different functional genes between the polyphagous and oligophagous species.

## Results

### Transcriptomic and proteomic analysis

The results of RNA sequencing from *C. punctiferalis* and *C. pinicolalis* were a total of 203,131 assembled unigenes with a mean length of 1119 bp and N50 length of 1753 bp (Table 1). The total number of sequences detected by mass spectrometry of both unique spectra were 21,646, which represented 13,680 unique polypeptides, and matched 3728 proteins (Table 2). The total number of DEPs between *C. punctiferalis* and *C. pinicolalis* were 391. The raw reads of the *C. punctiferalis* and *C. pinicolalis* were available on the NCBI SRA database (Accession numbers: SRR12988915, SRR12988916, SRR12988917 and SRR12989228, SRR12989229, SRR12989230).

**Table 1:** Summary of assembled contigs and unigenes

Type (bp)	Contig	Unigene
Total number	257,639	203,131
Total length	241,804,378	227,279,444
Min length	201	201
Mean length	939	1119
Maximum length	25,005	25,005
N50	1638	1753
N90	355	469
DEGs	–	74,611

**Table 2:** Summary of iTRAQ metrics

Metrics	Number
Unique polypeptide	13,680
Unique spectra	21,646
Matched proteins	3728
DEPs	391

### Correlation analysis between DEGs and DEPs

**Fig. 1:**
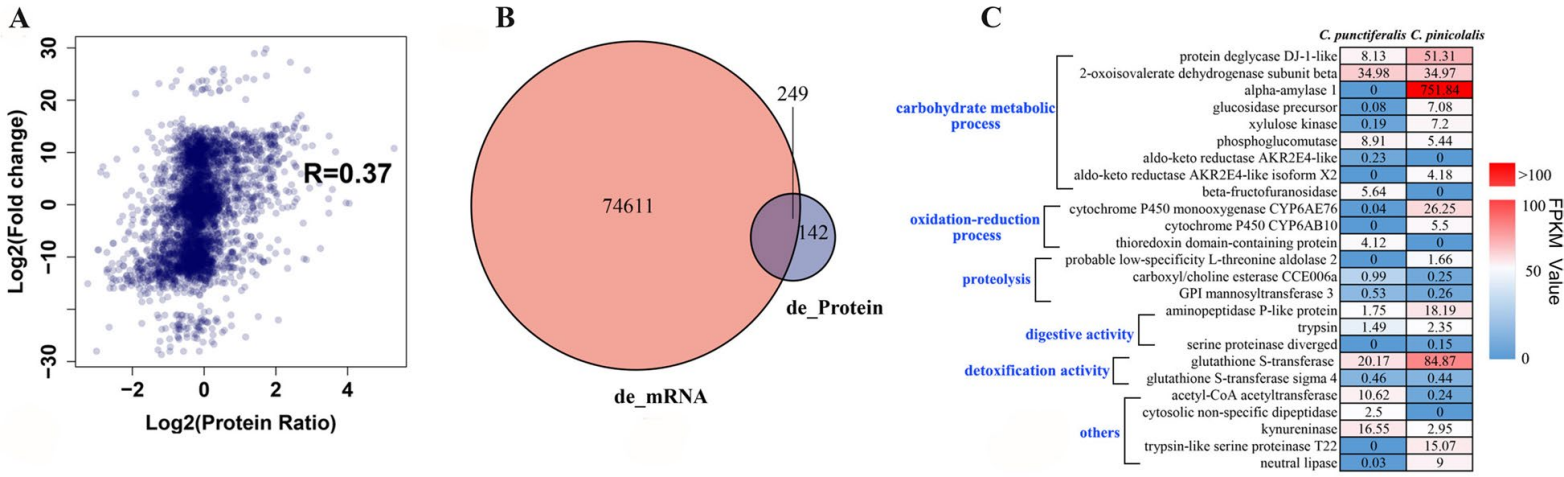
Interaction analysis of DEGs and DEPs. **A** Correction plot analysis based on DEGs and DEPs. **B** Correlation analysis of DEGs and DEPs depicted in the Venn diagram, 391 DEPs correlated with 74,611 transcripts. Out of 391, 249 transcripts and proteome data were overlapped, and 142 differential proteins were identified between the *C. pinicolalis* and *C. punctiferalis.* **C** Heat map based on FPKM value of DEGs and DEPs obtained from the samples of *C. pinicolalis* and *C. punctiferalis*

Totally, 74,611 DEGs and 391 DEPs were correlated and analyzed according to their difference multiples (Fig. 1A). GO analysis was used to classify the Biological process, Molecular function and Cellular components after the transcriptomic and proteomic correlate analysis (Additional file 1: Fig. S1A). The results showed that these enriched genes were mainly closely related to metabolism and redox (Fig. S1B and C). From KEGG annotation, the data is mainly closely related to metabolism and redox (Additional file 1: Fig. S2). After correlation analysis, we found that 249 transcripts overlapped with the proteome data, and 142 differential proteins were identified after correlation analysis (Fig. 1B). Next, we selected all the proteins (30 proteins) related to digestion and metabolism from 142 different proteins for further study (Fig. 1C, Additional file 1: Table S1). 9 proteins were selected for the open reading frame (ORF) amplification by PCR based on the transcriptome data (Table 3).

**Table 3:** Summary of genes selected from DEGs and DEPs

Gene name	Sequences similarity rate (%)	Gene description	References
α-amylase	94	Hydrolyses alpha bonds of large, alpha-linked polysaccharides	(Janecek, 1994) [8]
acetyl-CoA acetyltransferase	100	Catalytic enzyme	(Wiedow et al., 1996; Wakazono et al., 1995) [9, 10]
glutathione S-transferase	100	Detoxification	(Tu and Qin, 1987; Ye et al., 2005) [11, 12]
chymotrypsin BII-like	100	Diverged evolution, including digestive process	(Danwattananusorn, et al., 2009) [13]
P450 monooxygenase CYP6AE76	92	Detoxification	(Waters et al., 1992; Chen and Li, 2007; Chung et al., 2009) [14–16]
pancreatic triacylglycerol lipase	100	Digestive enzyme	(Lowe, 2002; Whitcomb and Lowe, 2007) [17, 18]
cytochrome P450 6B2	100	Detoxification cytochrome P450 6B2-like	(Waters et al., 1992; Chen and Li, 2007; Chung et al., 2009) [14–16]
beta-fructofuranosidase	100	Hydrolyze sucrose aiming to produce inverted sugar	(Schwebel-Dugue et al., 1994; Fouet et al., 1984) [19, 20]
protein dj-1beta-like	100	Antioxidants protein dj-1beta-like isoform X1	(Shendelman et al., 2004; Clements et al., 2006; Richarme et al., 2006) [21–23]

### Superimpositions and sequence comparison

**Fig. 2:**
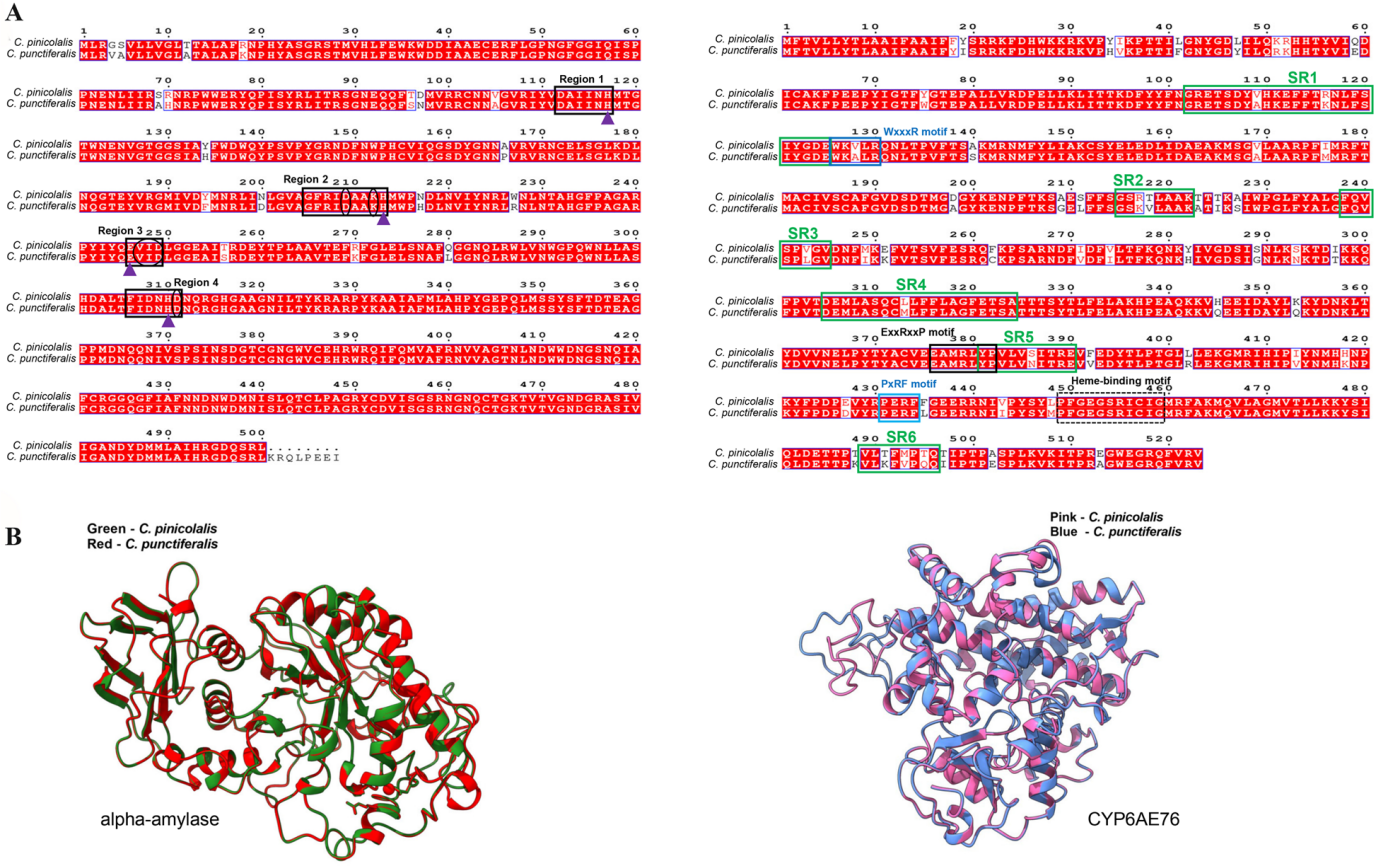
Diagram of the secondary and tertiary structure of α-amylases and CYP6AE76 amino acides. **A** Comparison of amino acid sequences of α-amylases and CYP6AE76. Homologous sequence regions 1, 2, 3 and 4 are surrounded by purple rectangles. The amino acid sequence in the rectangular was taken as representative of regions 1 to 4, respectively. Active sites and those of substrate binding proposed by Matsuura et al. [24] for Taka-amylase A from are indicated by the purple triangle and black oval, respectively; SRS is represented in green line boxes, heme-binding signature motif (FxxGxxxCxG) (blackdotted rectangular frame), helix C motif (WxxxR) (navy blue box), and PxPF motif are in lightboxes. **B** superimpositions of predicted models of α-amylase and CYP6AE76 from *C. pinicolalis* and *C. punctiferalis* with their respective templates

The ESPript 3.0 was used to compute the structure elements, α-amylase was highly conserved, and no amino acid mutations was found in the homologous sequence regions and active sites (Fig. 2A-left). CYP6AE76 had high sequence identities in six substrate recognition sites (SRS), including SRS1, SRS3, SRS4 and SRS5, but SRS2 and SRS6 showed the lowest identity. In addition, two sequences had mutations at the WxxR site, but not at either ExxRxxP or Heme-binding sites (Fig. 2A-right). Superimpositions of each model with the template using UCSF ChimeraX v1.1 software showed a very low RMSD value of 0.130 Å for α-amylase 1 from *C. pinicolalis* and *C. punctiferalis* (Fig. 2B). Similarly, a low RMSD value of 0.224 Å was observed between the superimposed CYP6AE76 3D structures of *C. pinicolalis* and *C. punctiferalis*, respectively (Fig. 2B). In addition, the 3D diagram indicated that partially differences of amino acid sequence did not affect the overall structural change.

### Detection of *α-amylase* and *CYP6AE76* expression levels

**Fig. 3:**
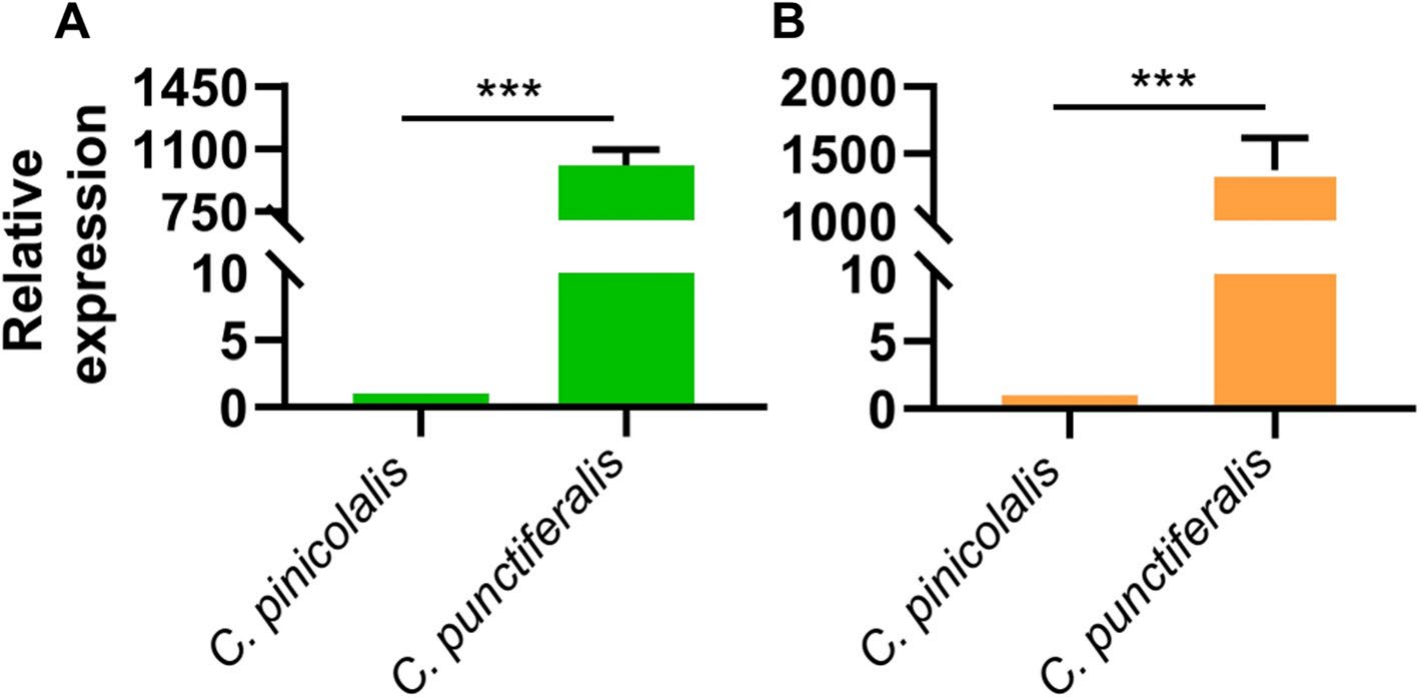
Gene expression of α-amylase and P450 monooxygenase CYP6AE76 in *C. pinicolalis* and *C. punctiferalis*. **A** The relative expression of α-amylase in *C. pinicolalis* and *C. punctiferalis*. **B** The relative expression of P450 monooxygenase *CYP6AE76* in *C. pinicolalis* and *C. punctiferalis*. The gene expression level between the two species was statistically significant (*t*-test, ***P < 0.001)

The DEGs and DEPs results showed the divergence of sequence similarity (Table 3). Importantly, *α-amylase* and P450 monooxygenase *CYP6AE76* showed 94% and 92% similarities between the two species, respectively. Therefore, both genes were selected for further study. The qPCR results of *α-amylase* and *CYP6AE76* showed significantly higher expression in *C. punctiferalis* compared with *C. pinicolalis* (Fig. 3). This result was also consistent in the transcriptome data (Fig. 1C).

### α-Amylase and CYP6AE76 activity

**Fig. 4:**
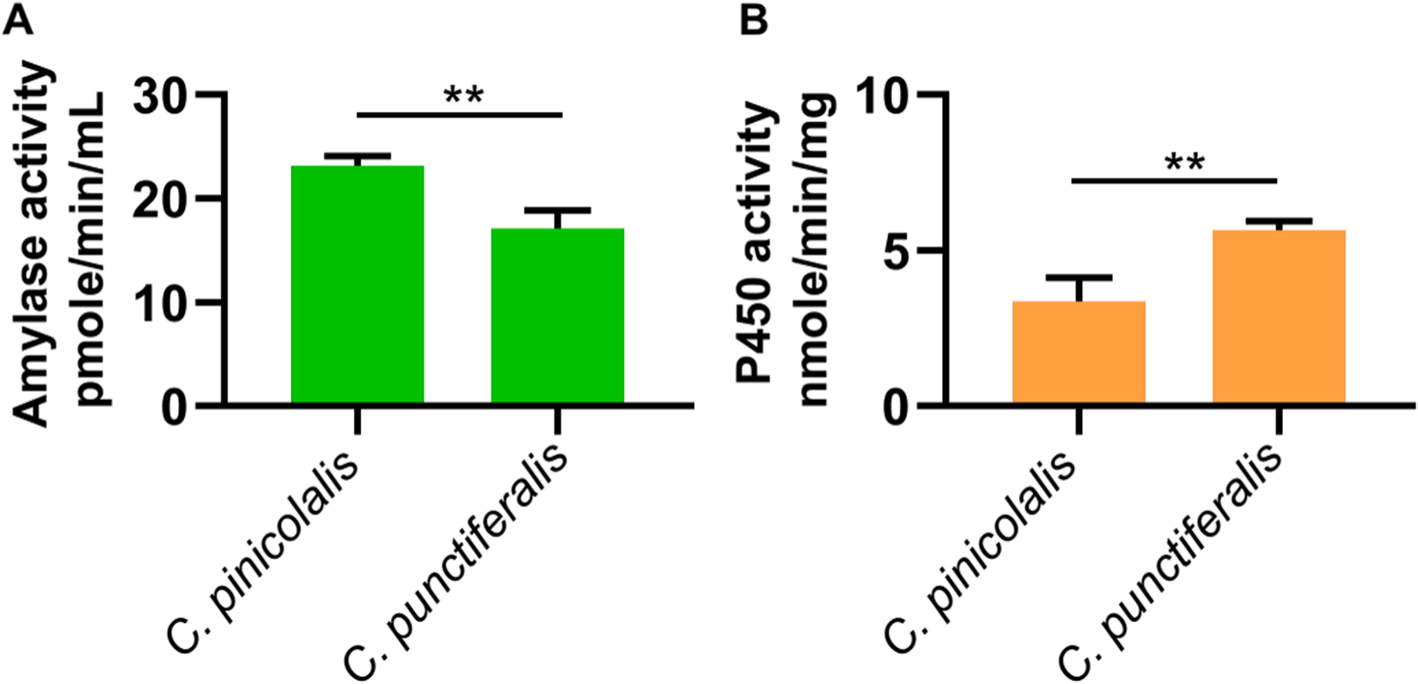
The comparison of enzymatic activity of recombinant α-amylase and CYP6AE76 from two species. **A** The amount of ethylidene-pNP-G7 (substrate) cleaved by the purified α-amylase from *C. pinicolalis* and *C. punctiferalis*. **B** Conversion of p-nitroanisole to p-nitrophenol by recombinant cytochrome P450 from *C. pinicolalis* and *C. punctiferalis*. The α-amylase and CYP6AE76 enzyme activities were statistically significant (Student’s *t*-test, **P < 0.01)

Four proteins were successfully expressed in *E. coli* (Additional file 1: Fig. S3 and S4), and tested obtained the enzyme activity after protein purification. The amount of substrate, ethylidene-pNP-G7, cleaved by the purified α-amylase from *C. pinicolalis* showed higher activity than *C. punctiferalis* (Fig. 4A). Furthermore, the conversion of p-NA to p-NP by CYP6AE76 was significantly higher in *C. punctiferalis* (Fig. 4B).

### Differentially changed metabolites in two species

**Fig. 5:**
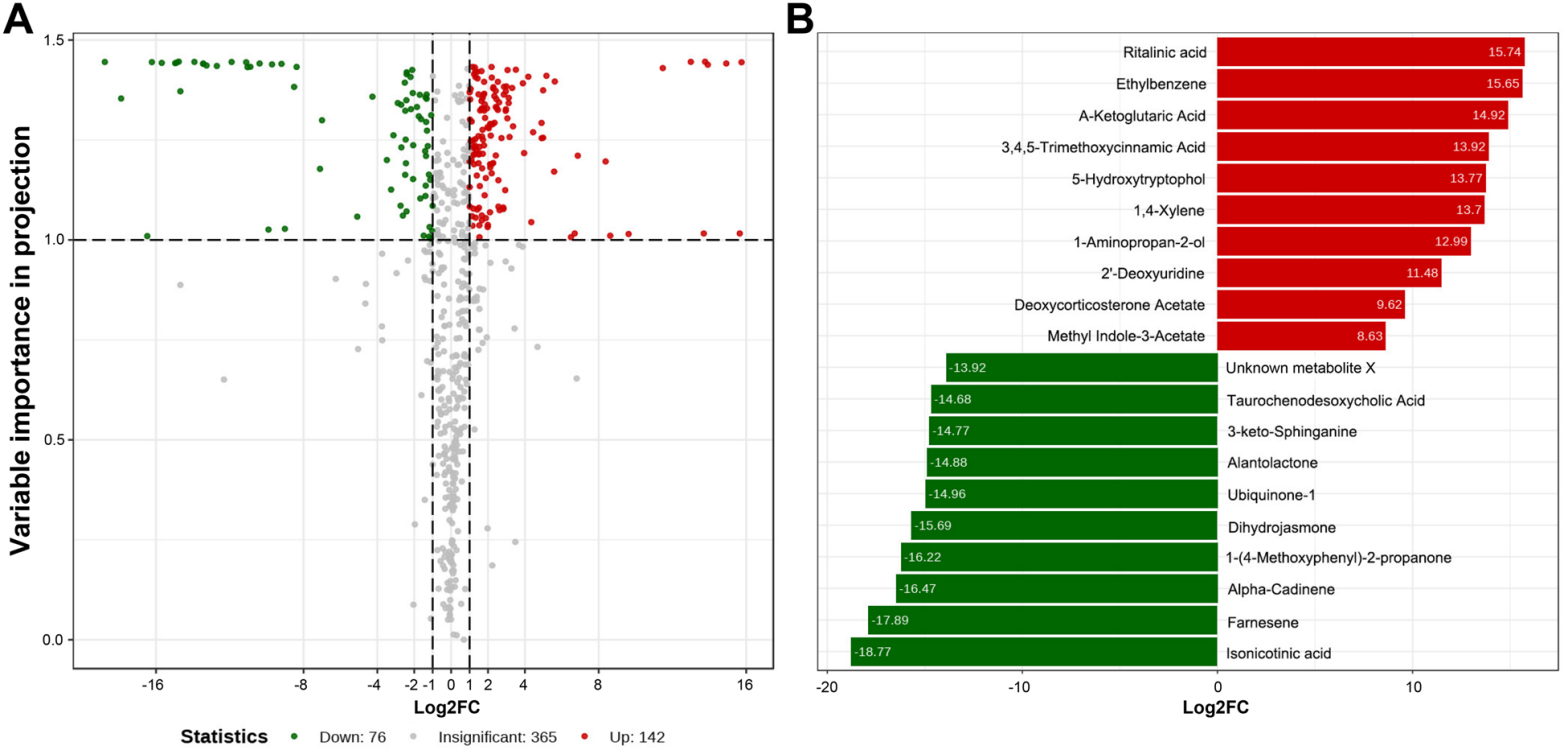
Association analysis of the different genes between the two species (*C. pinicolalis* vs. *C. punctiferalis*). **A** Volcano map of all differential expression genes; **B** Top 20 down- and up-accumulated metabolites between the two species

**Fig. 6:**
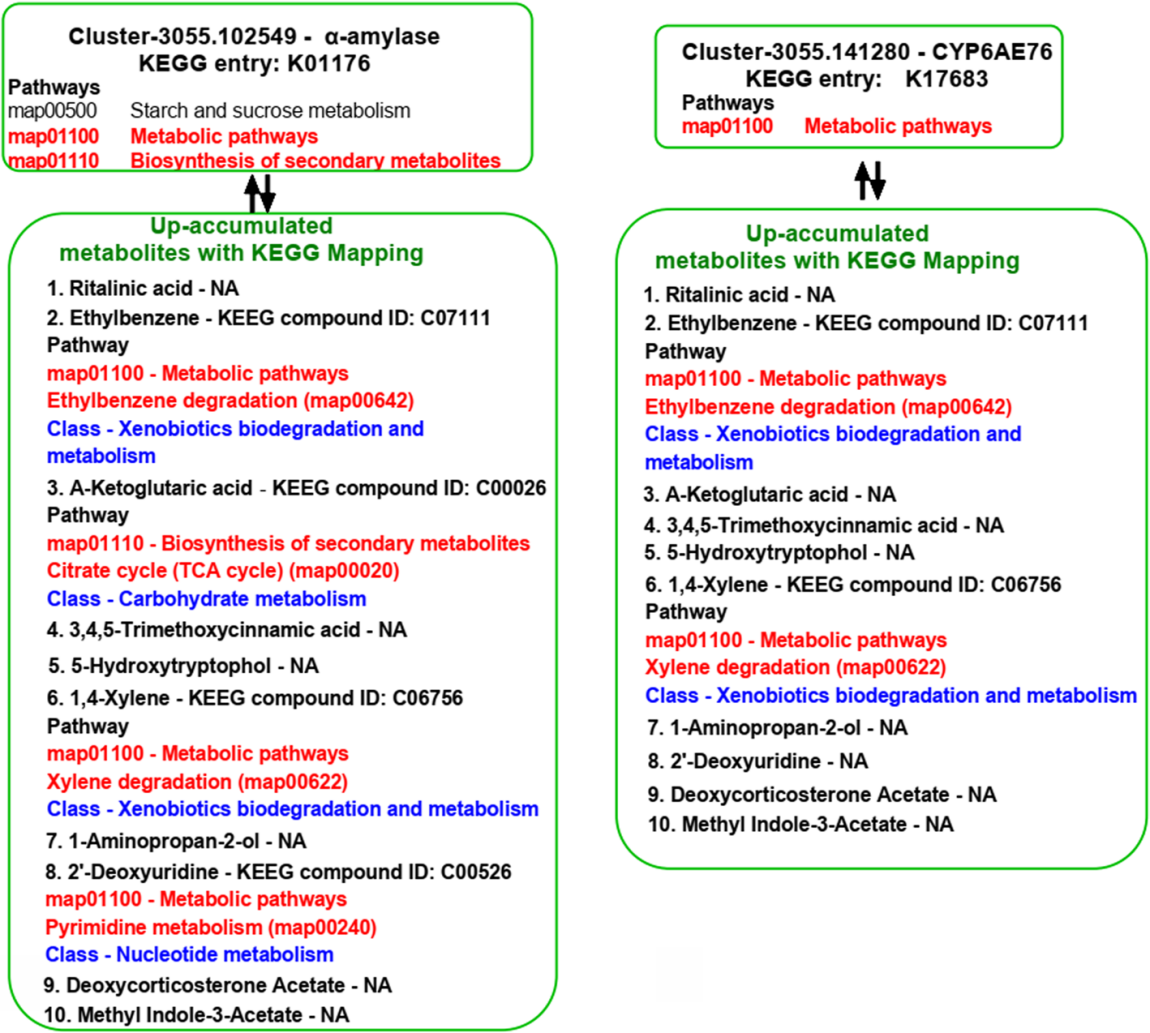
KEGG pathway mapping of α-amylase and P450 monooxygenase CYP6AE76 with the up-accumulated metabolites (depicted in Fig. 5) from the samples of *C. punctiferalis* compared to *C. pinicolalis*. The up-accumulated metabolites were statically significant (**P < 0.01). The words highlighted with red color represent the mapped KEGG pathway entry numbers associated with the identified metabolites with the compound identity numbers. Blue-colored words indicate the class and its general descriptions. NA - KEGG pathway not available

To further understand the difference in metabolism in two polyphagous and oligophagous species, we compared the metabolites in *C. punctiferalis* and *C. pinicolalis*. By comparative metabolome analysis, 583 differential expression genes were annotated (76 down and 142 up were accumulated, Fig. 5A), and the top 20 down- and up-accumulated differential metabolites were shown in Fig. 5B. From KEGG annotation, the data was mainly related to metabolic pathways and biosynthesis of secondary metabolities (Additional file 1: Fig. S5 and S6). The *α-amylase* and P450 monooxygenase *CYP6AE76* gene expression and the relation with up-accumulated metabolities was integrated with identified KEGG compound identity number with the respective pathways (Fig. 6).

### Levels of glutathione s-transferase and cytochrome P450 reductase

**Fig. 7:**
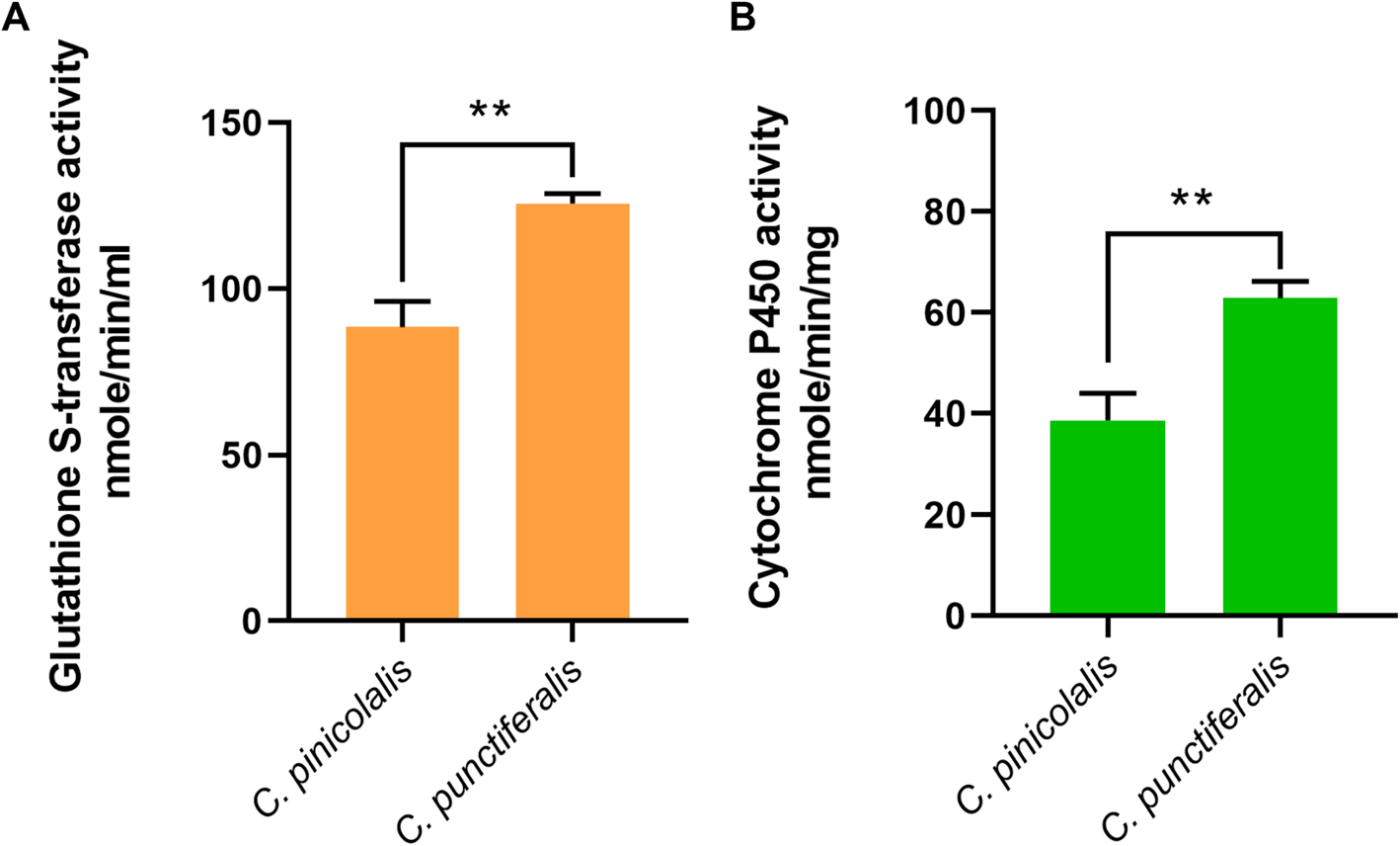
Assessment of glutathione S-transferase and cytochrome P450 reductase activity from total protein extracted from fourth instar whole larval body of *C. pinicolalis and C. punctiferalis*. **A** The amount of glutathione s-transferase was estimated from the samples of *C. pinicolalis* and *C. punctiferalis*. **B** Conversion of p-nitroanisole to p-nitrophenol by total cytochrome P450 from samples of *C. pinicolalis* and *C. punctiferalis*. The glutathione s-transferase and cytochrome P450 reductase enzyme activities were statistically significant (Student’s *t*-test, **P < 0.01)

The glutathione s-transferase (GST) activity from the whole larval body sample of *C. punctiferalis* showed significantly higher activity than the *C. pinicolalis* (Fig. 7A). Similarly, the cytochrome P450 reductase (CPR) was also observed to be significantly higher in *C. punctiferalis* than the *C. pinicolalis* (Fig. 7B).

## Discussion

The study of the multiple-omics techniques of polyphagous *C. punctiferalis* and oligophagous *C. pinicolalis* initially, from a particular aspect, revealled the potential mechanisms for the dietary differentiation of the two species. Our study found that 74,611 DEGs and 391 DEPs were correlated with their difference multiples (Fig. 1A). Further in correlation analysis, we found that 249 of the transcripts and proteome data were overlapped, and 142 differential proteins were identified between the *C. punctiferalis* and *C. pinicolalis* (Fig. 1B). Differential gene expression or mutation was a significant contributor to their disparate feeding habits. The polyphagous *C. punctiferalis* relies on multiple hosts, and they have different dietary habits than *C. pinicolalis.* Carbohydrates are required by both insect larvae and adults for energy demands, growth, longevity, movement, and reproduction [25, 26]. Similarly, plant defenses can occur at various times, including before ingestion, in the digestive tract prior to absorption, and within cells afterward [27, 28]. In insects, the midgut is the primary site of digestion and a key interface for plant allelochemical detoxification [29]. Phenolic compounds which are smaller in size may be able to cross the peritrophic membrane and directly cause lesions and oxidative stress in cells [30]. In some species, gene duplication or amplification has been shown to play a role in resistance or detoxication, and both improve the production of metabolic enzymes [31, 32]. The increased production of metabolic enzymes can break down or bind (sequester) to the pesticide [32].

Our study focused on digestive and detoxification-related genes and the sequence similarity between the polyphagous *C. punctiferalis* and oligophagous *C. pinicolalis*. Most of the selected digestive and detoxification-related genes DEGs and DEPs showed 100% sequence similarity, but not *α-amylase* and P450 monooxygenase *CYP6AE76* in our case (Table 3). The divergence in the two genes by mutation might be a potential reason for the species to adapt diatary changes. α-amylases improve the digestive performance of insects, allowing them to survive in different environments and increasing their biological fitness [33]. *α*-amylase is also an endoglycosidase enzyme to cleave an internal glycosidic bond within a poly (starch) or oligosaccharide (glycogen) and help form simple sugars like glucose (monosaccharide) and maltose (disaccharide) for energy. The *C. punctiferalis* has a high level of α-amylase expression and may have a link with the hosts. *C. punctiferalis* larvae have been reported to attack more than 100 essential plant species, including peach, durian, chestnut, citrus, papaya, cardamom, ginger [7], those of which can provide more carbohydrates. On the contrary, the host of *C. pinicolalis* is only Masson pine, the needles of which contain a lot of cellulose, fat and protein, etc. [34, 35]. Therefore, the type of food is relatively simple, its nutrients are limited, and the demand for amylase would be relatively low. In this study, the differential expression of the two genes in *C. punctiferalis* and *C. pinicolalis* is more closely related to their dietary habits.

The genomes of phytophagous insects usually contain large numbers of P450s, especially within the CYP3 clan. CYP6 subfamily members help detoxify plant host secondary metabolites [36–38]. Knockout of the CYP6AE cluster does not affect the viability of the insect, but it results in increased susceptibility to both plant toxins and synthetic insecticides [39]. As a polyphagous insect, *CYP6AE76* gene is not only highly expressed in larvae of *C. punctiferalis* (Fig. 3), but the enzyme activity level is also significantly higher than oligophagous *C. pinicolalis* (Fig. 4). Moreover, Mittapelly et al. [37] reported that the *CYP6* gene expression in polyphagous insects is not based on the host diet. However, they might apply a cocktail of broad-spectrum detoxification enzymes that interact with a variety of compounds in their diets, and these *CYP6Bs* may be part of the cocktail. Those results showed that polyphagous *C. punctiferalis* needs more *CYP6AE76* to metabolize or detoxify substances from a variety of foods. On the contrary, oligophagous *C. pinicolalis* only eats pine needles, so the need for multiple detoxification and metabolism might be low compared to *C. punctiferalis*. In addition, previous studies showed the induction of some *CYP6AE* genes by specific chemicals or different host plants [40, 41]. However, the pine needles may contain a small amount of specific chemical substances mentioned above, and the food source of *C. pinicolalis* is relatively single, resulting in low expression of the *CYP6AE76* gene.

Environmental conditions are not always suitable for survival, and insects employ multiple strategies for adaptation [42]. After long-term evolution, *C. punctiferalis* and *C. pinicolalis* have become distinct and distinguished by the mitochondrial cytochrome c oxidase gene [43, 44]. In this study, α-amylase and CYP6AE76 were found to have mutations after a multi-omics joint analysis. However, no mutations were detected in the homologous conserved regions, and enzyme active sites in *α-amylase* (Fig. 2A) with 94% sequence similarity (Table 3). On the contrary, mutations appeared in some other regions. Although these mutations do not cause structural changes, they may also differ significantly in their exact substrate preference and product profile [45]. Therefore, those mutations may have caused the high expression of α-amylase and its enzyme activity in *C. pinicolalis*, affecting their metabolism or detoxification of food afterward. Although the P450 superfamily has a wildly divergent sequence and the overall homology may be less than 40% even within the same family, particularly in insects [46], function-critical sequence motifs are preserved during the evolution of heme-binding sequence motif (FxxGxxxCxG) universal among CYP enzymes. In this study, no mutations in the heme-binding site were detected, suggesting no main functional change. However, all SRS sites of the two species have mutations. Amino acids in SRSs have been shown to affect the protein folding and substrate range of cytochrome P450s, particularly the SRS1 loop area near the heme active site, which significantly impacts various P450 functions [47–49]. Recently, Zuo et al. [50] revealed that the mutation in the SRS1 region of CYP9A186 of *Spodoptera exigua* causes resistance to both emamectin benzoate and benzoate abamectin. In addition, target-site resistance involves alterations (e.g., mutations) in the insecticide target protein that reduce its sensitivity to insecticides [51]. Therefore, mutation causing different binding ability could indirectly lead to the different de-toxification ability that has been verified by the qPCR and enzyme activity test. However, genetic mutation is one of many factors to affect feeding habits of insects, their long-term adaptation to the environment could select a broad range of genes in turn.

Metabolites are the final products of cellular regulatory processes. Therefore, it is necessary to understand the final metabolites difference in dietary habits between the two species. More metabolic difference substances are found in *C. punctiferalis* than in *C. pinicolalis*. Among the top 20 down and up accumulated metabolites, *C. punctiferalis* is mainly metabolized of amino acids, organic acids, and alcohols, while *C. pinicolalis* mainly metabolizes lipids, organic acids, and terpenes (Fig. 5B). These differences can also reflect their different foods resource, especially in *C. pinicolalis*. The pine needles contain many lipids [34], so these enzymes are needed for metabolism. This may also be the reason why its α-amylase activity is stronger than *C. punctiferalis*. The KEGG classification indicates that *C. punctiferalis* is enriched more in metabolic pathways than *C. pinicolalis*. For example, biosynthesis of amino acids, pyrimidine metabolism, ATP binding cassette transporters (ABC) transporters, etc. (Additional file 1: Fig. S4). However, ABC as a transporter has been increasingly recognized with resistance to cancer chemotherapy in humans, drug resistance in protozoa, antibiotic resistance in bacteria, and pesticide detoxification in nematodes, arthropods and Lepidoptera pests in recent years [52–54]. Additionally, KEGG pathway mapping of *α-amylase* and P450 monooxygenase *CYP6AE76* with the up-accumulated metabolites strengthens our research in terms of associated metabolome with KEGG pathways. On the mapping of *α-amylase* and P450 monooxygenase *CYP6AE76,* the genes were widely mapped with two pathways metabolic pathway and biosynthesis of secondary metabolites (Fig. 6). However, the mapped pathways in *C. punctiferalis* and identified metabolites were involved in carbohydrate metabolism and the xenobiotic biodegradation process (Fig. 6). The up-accumulated metabolite data and KEGG pathway mapping of highly expressed *α-amylase* and P450 monooxygenase *CYP6AE76* genes suggest that the polyphagous *C. punctiferalis* can interact the different plant compounds and nutritions without any fitness costs.

Toxic allelochemicals or xenobiotics from the leaves of the various host plants consumed by the insects have developed several enzymes, including cytochrome P450s, GSTs and esterases, that are involved in the detoxification process [55]. In our study, the whole larval body sample extracted from *C. punctiferalis* showed a significantly higher level of GST and CPR activities (Fig. 7 A and B). The larvae collected from the field and *C. punctiferalis* were reared on different diets, and *C. pinicolalis* was reared on Masson pine branches. The final GST and CPR enzyme assays revealed the polyphagous *C. punctiferalis* with elevated levels of GST and CPR enzyme activities, then the monophagous *C. pinicolalis.* In contrast, the DEG and DEP results showed a higher expression of GSTs and CPRs in *C. pinicolalis* (Fig. 1 C) and *C. punctiferalis* larvae collected from the Langfang Experimental Station were used to prepare samples as well as for DEGs and DEPs analysis. The *C. punctiferalis* larvae greatly depend on maize as a host plant due to its abundance. Larvae feeding on a single host plant may be an important reason that the larval system does not need to handle more xenobiotics, resulting in less expression of GSTs and CPRs. However, our GST and CPR experiments revealed that when the larvae reared on different host plants, it enhanced the detoxification process. A CPR class enzyme CYP6B8 in polyphagous pest *Helicoverpa zea* can metabolize six plant allelochemicals [56]. In *Spodoptera litura*, the glutathione S-transferase epsilon 1 gene in the midgut was highly expressed after exposure to host phytochemicals indole-3-carbinol and allyl-isothiocyanate and suggested glutathione S-transferase epsilon 1 critical detoxifying protein it may be related to host plant adaptation [57]. Finally, in our study, the KEGG pathway mapping of α-amylase and P450 monooxygenase *CYP6AE76* revealed the up-accumulatio of metabolites in *C. punctiferalis* (Fig. 5). The mapped KEGG revealed the pathway functions connected to xenobiotic biodegradation and metabolism (Fig. 6). All the results suggest the polyphagous pest may use different detoxification systems enzymes to adapt to a wide range of plants. An insect’s adaptation or preference to a wide range of host plants is the result of long-term evolution between the pest and its host plants.

## Conclusion

To summarize, the present study showed that the mRNA levels, proteins and metabolites had significantly altered in polyphagous *C. punctiferalis* and oligophagous *C. pinicolalis* by the multi-omics techniques, and all the data were mainly closely related to metabolism and redox. In particular, the *α-amylase* and *CYP6AE76* gene mutations lead to differences in gene expression levels and enzyme activities, resulting from a long-term evolutionary selection between the two species. These findings will offer new perspectives for understanding the molecular mechanisms of polyphagous and oligophagous insects.

## Methods

### Insects rearing and antennae collection

*C. punctiferalis* larvae were collected from corn ear at Langfang Experimental Station of Chinese Academy of Agricultural Sciences, Hebei Province, China, and reared on fresh corn ear in an environmentally controlled room at 27 ± 1 °C, 70–80% relative humidity (RH), and 16:8 light: dark (L:D).

*C. pinicolalis* larvae were collected from the Masson pine in Quanjiao County (32.07 N 117.54 E), Anhui Province, China. Fresh Masson pine branches were used to feed the larvae under ambient conditions 27 ± 0.5 °C, with 70–75% relative humidity (RH) and a photoperiod of 16:8 h light: dark (L:D). After emergence, the moths were fed on a 10% honey solution [58].

### RNA extraction and transcriptome sequencing

Total RNA was extracted from the whole larval body of *C. punctiferalis* and *C. pinicolalis* fourth instar larvae using the Quick-RNA MicroPrep Kit (ZYMO Research, USA) according to the manufacturer’s protocol. Three biological replicates were maintained (1 larva/replicate). The integrity of the total RNA was analyzed using 1.5% agarose gel electrophoresis [59]. The quality and concentration were analyzed on NanoDrop 2000 spectrophotometer (Thermo Scientific, USA). According to their instructions, the cDNA was synthesized using RT™ All-in-One Master Mix Kit (Herogen Biotech, USA). Transcriptome sequencing was performed at Novogen Co., Ltd. Beijing, China, and the samples were sequenced on the Illumina Hiseq 2500 platform. The raw reads were curated by removing adaptor sequences and reads of low quality, then assembled into unigenes using Trinity [60, 61].

### Protein extraction and sequencing

Total proteins were extracted from the fourth instar whole larval body of *C. punctiferalis* and *C. pinicolalis* with three biological replicates (1 larva/replicate) using a previously described protocol [62] with minor modifications. Samples were ground to a powder with liquid nitrogen and lysed with 2 mL lysis buffer containing 8 M urea, 2 M thiourea, 0.1% 3-[(3-cholamidopropyl) dime-thylammonio propanesulfonate (CHAPS) (Amresco Ltd., USA) and 1× Protease Inhibitor Cocktail (Roche, USA). The lysis solution was centrifuged at 4 °C, 13,000×g for 15 min to collect the supernatant in a new tube and then saved at − 80 °C until use. The protein concentration was determined using a 2-D Quant Kit (GE Healthcare, USA), and quality was examined with SDS-PAGE (Beyotime, China). Protein digestion was conducted using trypsin (Promega, USA) at 37 °C overnight, and peptides were dried in a centrifugal vacuum concentrator.

According to a previously described protocol, protein isolation and labeling were performed using the 8-plex iTRAQ (Applied Biosystems) according to a previously described protocol [63] with some modifications. Sample peptides were subjected to nano-electrospray ionization, followed by tandem mass spectrometry (MS/MS) in an Or-bitrap Q-Exactive plus system (Thermo Fisher Scientific, USA). MS scans were obtained from m/z 350–1800, with 40 precursors selected for MS/MS from m/z 100–1800 using a dynamic exclusion of 40 s for the selected ions. The collision-induced dissociation (CID) energy was automatically set as 32%. The database search strategy-based peptide matching tolerance was controlled below 10 ppm and 0.05 Da to prevent the omission of proteins.

### Metabolomics analysis

MetWare (Wuhan, China) performed the extracted analysis, metabolite identification, and quantification following their standard procedures and a previous study [64]. The fourth instar larvae of *C. punctiferalis* and *C. pinicolalis* were snap-frozen in liquid nitrogen for 5 minutes, grounded into fine powder in liquid nitrogen, and freeze-dried for 24 h. Ten snap-frozen larvae were grounded together to make one replicate, and a total of five biological replicates were maintained for both the species. The freeze-dried powdery samples (50 mg) were weighed and transferred to a 2 mL microcentrifuge tube and 1 mL pre-cooled methanol aqueous extractant (70%) containing a standard internal L-2-Chlorophenylalanine (1 μg/mL) was added to each tube. Pre-cooled small steel balls were added to each tube and homogenized for 3 min at 30 Hz in an ultrasonicator, the steel balls were removed from the tube and vortexed for 1 min, and the tubes were placed on ice for 15 min. Samples were centrifuged (13,000×g, 4 °C for 10 min), and 250 μL of supernatant from each tube were collected using syringes and filtered through microfilters (0.22 μm pore size). The filtered supernatants (about 150 μL) were transferred into an LC vial and stored at − 80 °C for further analysis. The samples were analyzed using Ultra Performance Liquid Chromatography (UPLC) (Shim-pack UFLC Shimadzu, CBM30A, Japan) data acquisition system with tandem mass spectrometry (MS/MS) (5500 QTRAP®, Sciex, MA, USA). The ACQUITY UPLC HSS T3 C18 (1.8 μm, 2.1 mm × 100 mm) (Waters, MA, USA) UPLC column analyzed the samples. The analytical conditions were as follows: Mobile phase A - Ultrapure water with 0.04% acetic acid, Phase B - acetonitrile with 0.04% acetic acid. Elution gradient were as follows: water/acetonitrile (95:5 V/V) at 0 min, 5:95 V/V at 11.0 min, 5:95 V/V at 12.0 min, 95:5 V/V at 12.1 min, 14.0 min is 95:5 V/V. The flow rate at 0.4 mL/min; column temperature 40∘ C; injection volume 2 μL. The mass spectrometry conditions mainly include electrospray ionization (ESI) temperature 500 °C, MS voltage 5500 V (positive), − 4500 V (negative), ion source gas I (GS I) 55 psi, gas II (GS II) 60 psi, curtain gas (CUR) 25 psi. The induced ionization (collision-activated dissociation, CAD) parameter was set to high. In the triple quadrupole (Qtrap), each ion pair is scanned and detected according to the optimized declustering potential (DP) and collision energy (CE). On the basis of the self-built target standard database MWDB (metware database), qualitative analysis was performed based on the retention time (Retention time) of the detected substances, information from the parent ion pair, and secondary spectrum data.

### Correlation analysis

Correlation analysis was carried out between differentially expressed genes (DEGs) and differentially expressed proteins (DEPs). Functional annotation of transcripts and proteins data were searched using BLASTX against the non-redundant (nr) NCBI protein database [65]. The calculation of unigene expression uses the FPKM method (Fragments Per kb per Million reads); In addition, using Blast2GO (http://www.blast2go.org) [66], we predicted and classified functions of unigenes by Clusters of EuKaryotic of orthologous groups (KOG) database [67]. In addition, the online Kyoto Encyclopedia of Genes and Genomes (KEGG) Automatic Annotation Server (KAAS) was employed for KEGG pathway enrichment analysis following the procedure of pathway annotations for transcripts and proteins data [24, 68].

### Gene sequences verify and qPCR detection

Total RNA was reverse transcribed to cDNA using RT™ All-in-One Master Mix Kit (Herogen Biotech, Shanghai, China) according to the protocol manufactures’ protocol, then PCR technology was used to amplify the selected gene sequences in two species. qPCR (quantitative real-time PCR) experiments were conducted with ribosomal protein RP49 as reference gene [69], and calculations were performed as described previously [70]. All primer sequences are given in supplementary file (Additional file 1: Table S3).

### Computational analysis of α-amylase and CYP6AE76

The amino acid sequences of α-amylase and cytochrome P450 (CYP) monooxygenase CYP6AE76 of *C. pinicolalis* and *C. punctiferalis* were submitted to structure homology modeling using Swiss-Model server (https://swissmodel.expasy.org/) [71]. The UCSF ChimeraX v1.1 was used to superimpose and visualize the 3D modeled structures of above two genes [72]. The ESPript 3.0 was used to compare the amino acid sequences of α-amylase and CYP6AE76 of *C. pinicolalis* and *C. punctiferalis* (https://espript.ibcp.fr/ESPript/ESPript/) [73].

### Preparation of recombinant protein

The methods of protein expression, purification and Western blot were followed by the previously reported [70]; more specific parameters were shown in Additional file 1 (Fig. S3 and Table S3).

### Enzyme activity assays

The α-amylase activity was tested using an amylase activity assay kit (Sigma-Aldrich, MO, USA) according to the manufacturer’s protocol. Briefly, 20 μL (0.5 mg/mL) of purified α-amylase protein expressed in the *Escherichia coli* system and 30 μL of Amylase assay buffer was added to each well of the microplate. The reaction was initiated by adding 100 μL of the Master reaction mix and mixed using a horizontal shaker. After 3 min, an initial optical density was read at 405 nm. The plate was incubated at 25 °C and measured the absorbance (405 nm) every 5 minutes. One unit is the amount of amylase that cleaves ethyli-dene-pNP-G7 to generate 1.0 μmole of p-nitrophenol (p-NP) per minute at 25 °C.

The CYP6AE76 activity was assessed according to the method reported by Qian et al. [74] and Shabbir et al. [75] with slight modification. A 125 μL of 2 mM p-nitroanisole (p-NA) solution and 50 μL (0.5 mg/mL) cytochrome P450 monooxygenase expressed in *E. coli* were added to each well of a microplate and mixed. This mixture was incubated at 27 °C for 2 min, and the reaction was initiated by adding 25 μL of 9.6 mM NADPH. The optical density at 405 nm was recorded using a microplate reader (FlexStation 3, Molecular Devices, CA, USA). Six replicates were maintained for α-amylase and CYP6AE76 enzyme activities.

### Glutathione S-transferase and cytochrome P450 reductase activity

*C. punctiferalis* and *C. pinicolalis* early second instar larvae were collected from the field and reared in laboratory conditions (section 5.1). The *C. pinicolalis* larvae were fed with fresh Masson pine branches, and the *C. punctiferalis* was fed fresh corn ear, peach fruit, peach leaves, and apple fruit in alternate feedings. The activities of glutathione s-transferase (GST) and cytochrome P450 reductase (CPR) were estimated from the fourth instar whole larval body of *C. pinicolalis* and *C. punctiferalis*. Total protein was extracted by homogenizing the larvae (*n* = 5) in a glass homogenizer containing 1 mL of ice-cold lysis buffer (50 mM Tris-HCl, 150 mM NaCl, 1% NP-40, 0.25% Na-deoxycholate, 1 mM N-Phenylthiourea, 1 mM Protease Inhibitor Cocktail (Roche, USA), pH 7.4). The homogenate was transferred to a microfuge tube, placed on ice for 10 min, and centrifuged at 10,000×g for 15 min at 4 °C, and the supernatant was used to estimate GST and CPR enzyme activities. The protein concentrations in the samples were estimated using the Easy Protein quantitative kit (TransGen Biotech Co., Ltd., Beijing, China), and the sample concentrations were set to 1 mg/mL before subjecting to GST and CPR enzyme activities. GST activity was measured using the Glutathione S-Transferase Assay Kit (Cayman Chemical, USA), according to the manufacturer’s instructions. Briefly, 20 μL of prepared sample was added to individual wells in a 96-well microplate. To the samples, 150 μL of assay buffer and 20 μL of glutathione provided with the kit were added per well. Finally, 10 μL of 1-chloro-2,4-dinitrobenzene (CDNB) was added, and the plate was incubated (10 min at 25 °C) and then read at 340 nm to measure the conjugation of CDNB with reduced glutathione.

The CPR activity was assessed using the method mentioned in section 5.9 to assess the CYP6AE76 activity. A 125 μL of 2 mM p-nitroanisole (p-NA) was added to the 96-well microplate, and a 50 μL total protein sample was extracted from larvae was added to the well-containing p-NA and mixed well. This mixture was incubated (2 min at 27 °C), and the reaction was initiated by adding 25 μL of 9.6 mM NADPH. The optical density at 405 nm was recorded using a microplate reader (FlexStation 3, Molecular Devices, CA, USA). Six replicates were maintained for GST and CPR enzyme activities.

## Supplementary Information

**Additional file 1.**

## Data Availability

All data generated or analyzed during this study are included in this published article and its supplementary materials. All Illumina data have been deposited in NCBI’s Sequence Read Archive (SRA) under accession number SRR12988915, SRR12988916, SRR12988917 and SRR12989228, SRR12989229, SRR12989230, and subsequent can be downloaded at https://www.ncbi.nlm.nih.gov/sra?linkname=bioproject_sra_all&from_uid=674682 and https://www.ncbi.nlm.nih.gov/sra?linkname=bioproject_sra_all&from_uid=674710.
