# Supplementary material for: Revealing the difference of α-amylase and CYP6AE76 gene between polyphagous Conogethes punctiferalis and oligophagous C. pinicolalis by multiple-omics and molecular biological technique
Source: BMC Genomics. 2022 Jul 19;23:521. doi: 10.1186/s12864-022-08753-9 (PMC9295484; doi:10.1186/s12864-022-08753-9)
Supplement: Supplementary file 1 — Additional file 1. [file 12864_2022_8753_MOESM1_ESM.pdf]

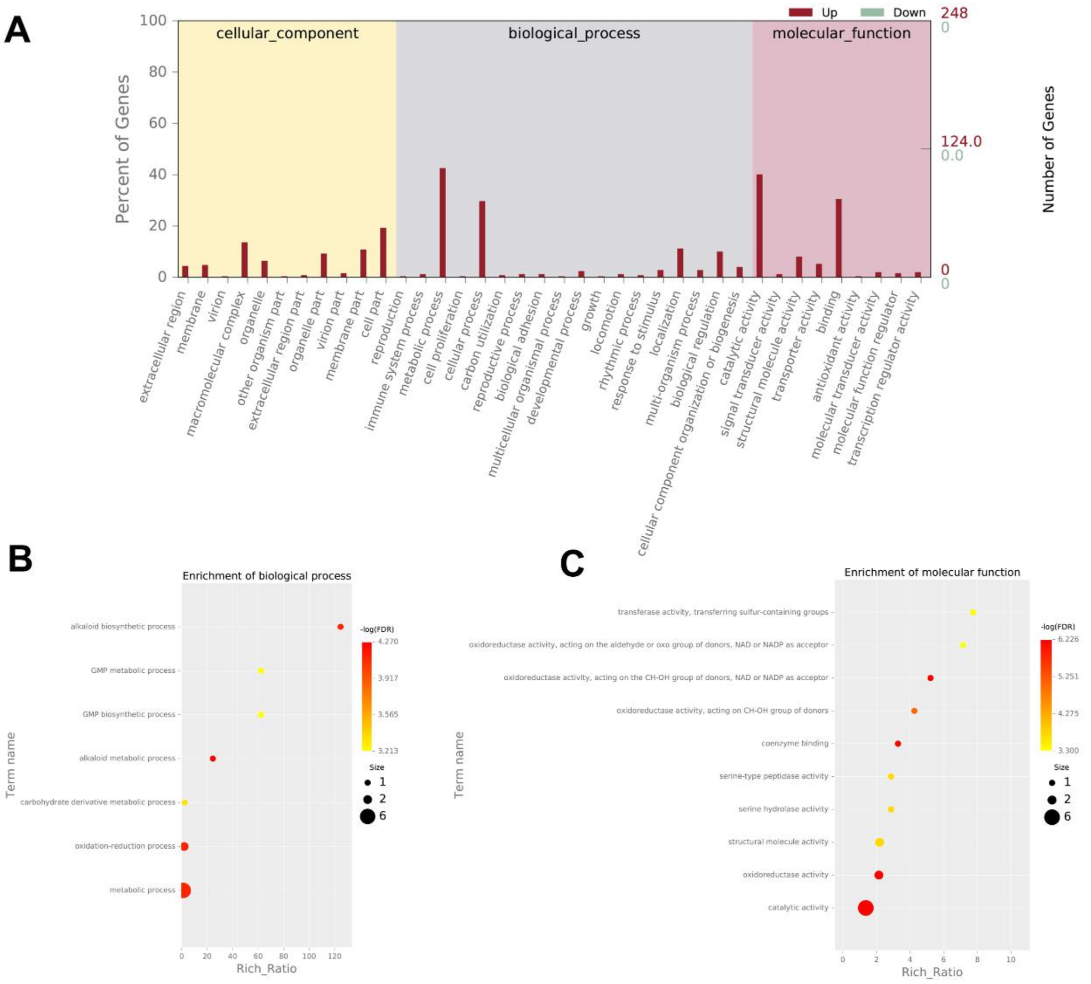

Figure S1. Transcriptome and proteomics association analysis. **(A)** Differential gene GO enrichment. **(B)** and **(C)** Differential gene GO enrichment of biological process and molecular function, respectively.

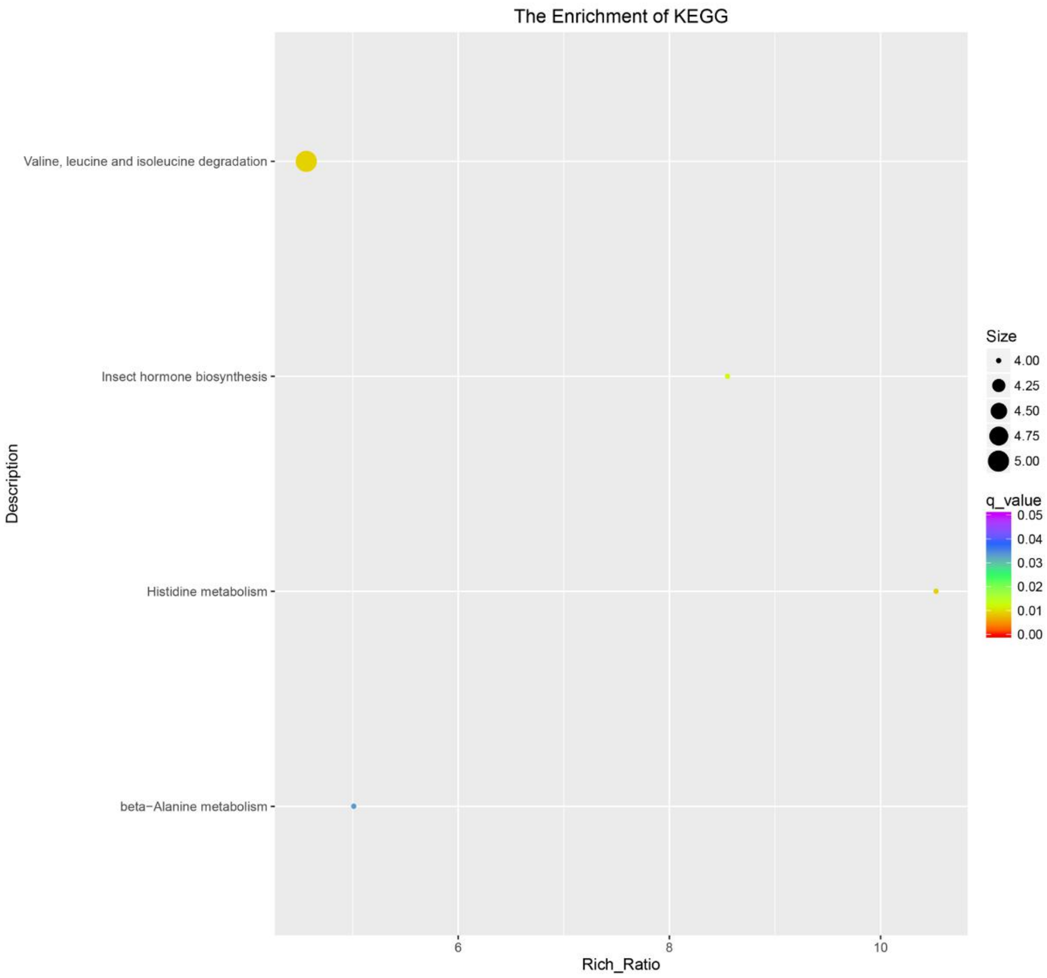

Figure S2. KEGG pathway enrichment scatter plot of the different genes.

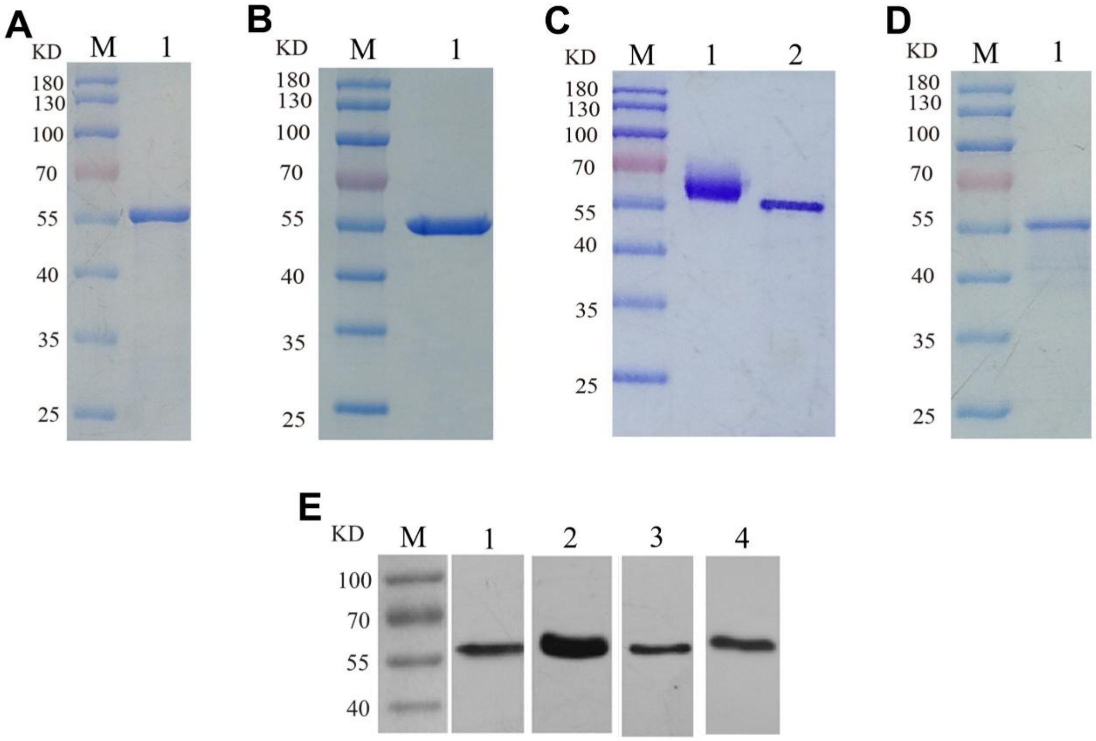

Figure S3. The SDS-PAGE gel figures of candidate genes were expressed and purified using *E. Coli.* (A) Lane 1: purified α-amylase recombinant protein of *C. pinicolalis*. (B) Lane 1: purified α-amylase recombinant protein of *C. punctiferalis*. (C) Lane 1: Bovine serum albumin and Lane 2: purified CYP6AE76 recombinant protein of *C. pinicolalis*. (D) Lane 1: purified CYP6AE76 recombinant protein of *C. punctiferalis*. M: Protein marker. (E) Western blot analysis of α-amylase and CYP6AE76 proteins. Lane 1 and 2: purified α-amylase proteins of *C. pinicolalis* and *C. punctiferalis*. Lane 3 and 4: purified CYP6AE76 proteins of *C. pinicolalis* and *C. punctiferalis*. M: Protein marker.

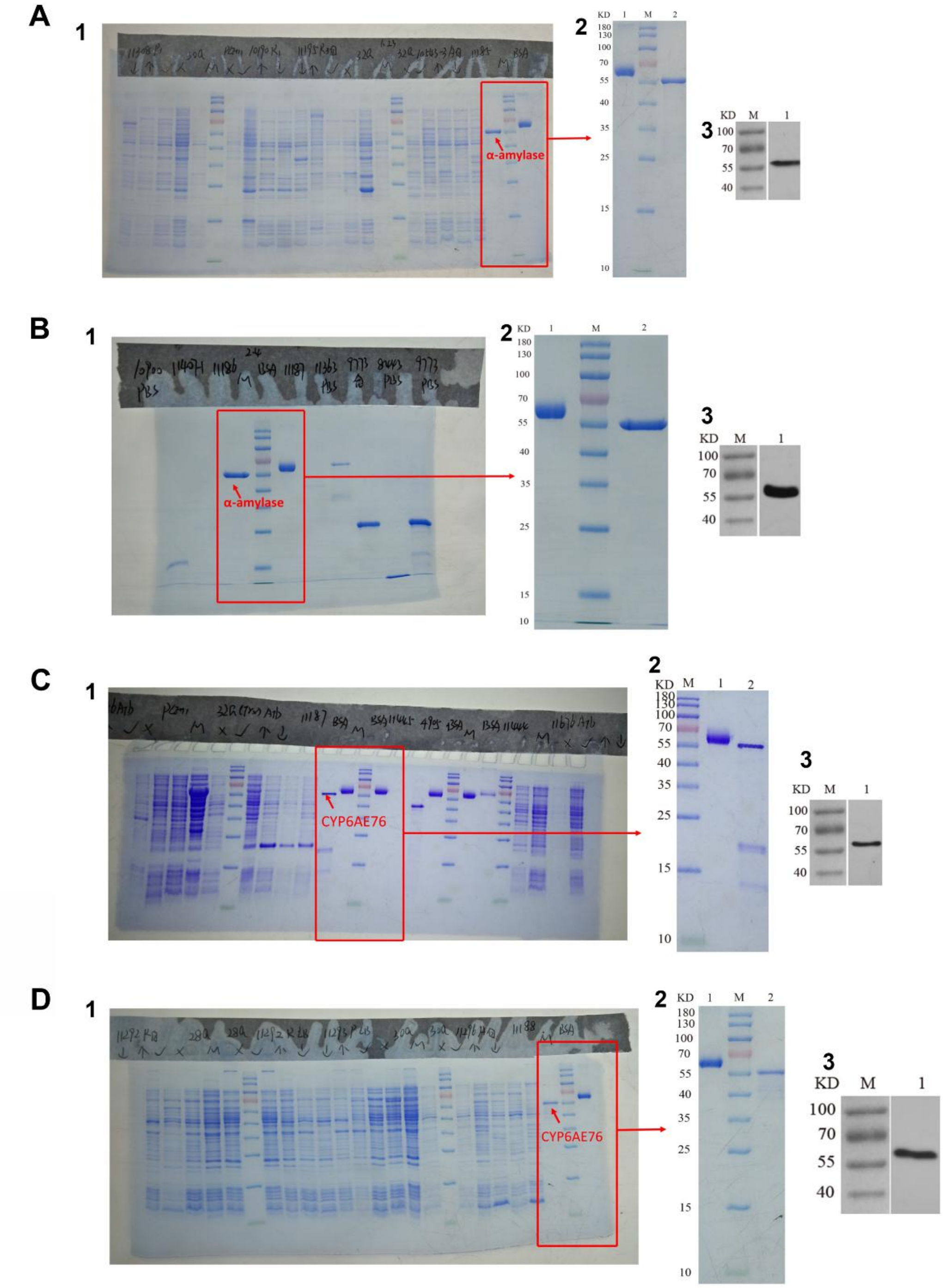

Figure S4. The uncropped SDS-PAGE figures of candidate recombinant protein. (A) and (B) α-amylase recombinant protein of *C. pinicolalis* and *C. punctiferalis*. (C) and (D) CYP6AE76 recombinant protein of *C. pinicolalis* and *C. punctiferalis.* Figure inset 1 indicates the uncropped SDS-PAGE gel, and the red box shows the area cropped and used for making supplementary figure S3. Inset 2: Cropped regions showing respective candidate recombinant proteins, Lane1: Bovine serum albumin and Lane 2: candidate recombinant protein. Inset 3: Western blot analysis of α-amylase and CYP6AE76 proteins of *C. pinicolalis* and *C. punctiferalis.* Lane 1 shows that candidate recombinant proteins lanes were excised from the SDS-PAGE gel and utilized for Western blot analysis, respectively. M: Protein marker.

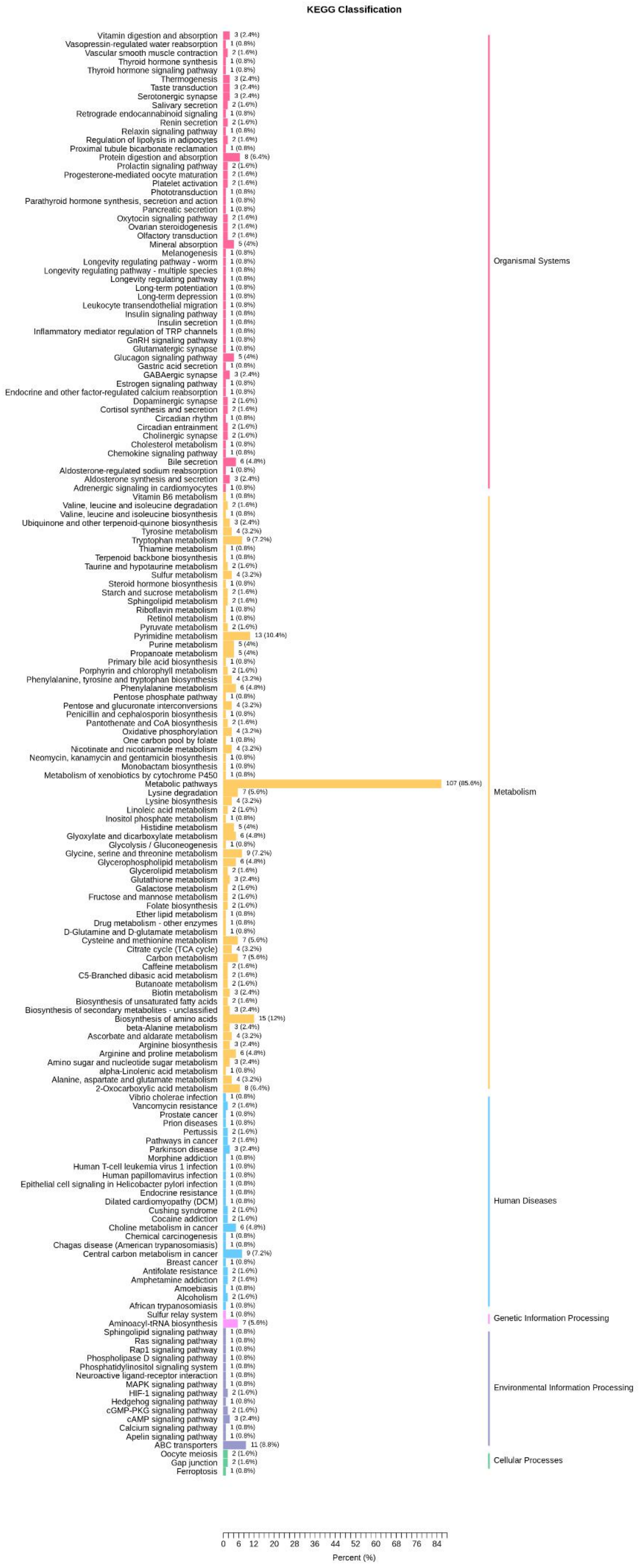
KEGG Classification
Vitamin digestion and absorption 3 (2.4%)
Vasopressin-regulated water reabsorption 1 (0.8%)
Vascular smooth muscle contraction 2 (1.6%)
Thyroid hormone synthesis 1 (0.8%)
Thyroid hormone signaling pathway 1 (0.8%)
Thermogenesis 3 (2.4%)
Taste transduction 3 (2.4%)
Serotonergic synapse 3 (2.4%)
Salivary secretion 2 (1.6%)
Retrograde endocannabinoid signaling 1 (0.8%)
Renin secretion 2 (1.6%)
Relaxin signaling pathway 1 (0.8%)
Regulation of lipolysis in adipocytes 2 (1.6%)
Proximal tubule bicarbonate reclamation 1 (0.8%)
Protein digestion and absorption 8 (6.4%)
Prolactin signaling pathway 2 (1.6%)
Progesterone-mediated oocyte maturation 2 (1.6%)
Platelet activation 2 (1.6%)
Phototransduction 1 (0.8%)
Parathyroid hormone synthesis, secretion and action 1 (0.8%)
Pancreatic secretion 1 (0.8%)
Oxytocin signaling pathway 2 (1.6%)
Ovarian steroidogenesis 2 (1.6%)
Olfactory transduction 2 (1.6%)
Mineral absorption 5 (4%)
Melanogenesis 1 (0.8%)
Longevity regulating pathway - worm 1 (0.8%)
Longevity regulating pathway - multiple species 1 (0.8%)
Longevity regulating pathway 1 (0.8%)
Long-term potentiation 1 (0.8%)
Long-term depression 1 (0.8%)
Leukocyte transendothelial migration 1 (0.8%)
Insulin signaling pathway 1 (0.8%)
Insulin secretion 1 (0.8%)
Inflammatory mediator regulation of TRP channels 1 (0.8%)
GnRH signaling pathway 1 (0.8%)
Glutamatergic synapse 1 (0.8%)
Glucagon signaling pathway 5 (4%)
Gastric acid secretion 1 (0.8%)
GABAergic synapse 3 (2.4%)
Estrogen signaling pathway 1 (0.8%)
Endocrine and other factor-regulated calcium reabsorption 1 (0.8%)
Dopaminergic synapse 2 (1.6%)
Cortisol synthesis and secretion 2 (1.6%)
Circadian rhythm 1 (0.8%)
Circadian entrainment 2 (1.6%)
Cholinergic synapse 2 (1.6%)
Cholesterol metabolism 1 (0.8%)
Chemokine signaling pathway 1 (0.8%)
Bile secretion 6 (4.8%)
Aldosterone-regulated sodium reabsorption 1 (0.8%)
Aldosterone synthesis and secretion 3 (2.4%)
Adrenergic signaling in cardiomyocytes 1 (0.8%)
Organismal Systems
Vitamin B6 metabolism 1 (0.8%)
Valine, leucine and isoleucine degradation 2 (1.6%)
Valine, leucine and isoleucine biosynthesis 1 (0.8%)
Ubiquinone and other terpenoid-quinone biosynthesis 3 (2.4%)
Tyrosine metabolism 4 (3.2%)
Tryptophan metabolism 9 (7.2%)
Thiamine metabolism 1 (0.8%)
Terpenoid backbone biosynthesis 1 (0.8%)
Taurine and hypotaurine metabolism 2 (1.6%)
Sulfur metabolism 4 (3.2%)
Steroid hormone biosynthesis 1 (0.8%)
Starch and sucrose metabolism 2 (1.6%)
Sphingolipid metabolism 2 (1.6%)
Riboflavin metabolism 1 (0.8%)
Retinol metabolism 1 (0.8%)
Pyruvate metabolism 2 (1.6%)
Pyrimidine metabolism 13 (10.4%)
Purine metabolism 5 (4%)
Propanoate metabolism 5 (4%)
Primary bile acid biosynthesis 1 (0.8%)
Porphyrin and chlorophyll metabolism 2 (1.6%)
Phenylalanine, tyrosine and tryptophan biosynthesis 4 (3.2%)
Phenylalanine metabolism 6 (4.8%)
Pentose phosphate pathway 1 (0.8%)
Pentose and glucuronate interconversions 4 (3.2%)
Penicillin and cephalosporin biosynthesis 1 (0.8%)
Pantothenate and CoA biosynthesis 2 (1.6%)
Oxidative phosphorylation 4 (3.2%)
One carbon pool by folate 1 (0.8%)
Nicotinate and nicotinamide metabolism 4 (3.2%)
Neomycin, kanamycin and gentamicin biosynthesis 1 (0.8%)
Monobactam biosynthesis 1 (0.8%)
Metabolism of xenobiotics by cytochrome P450 1 (0.8%)
Metabolic pathways 107 (85.6%)
Lysine degradation 7 (5.6%)
Lysine biosynthesis 4 (3.2%)
Linoleic acid metabolism 2 (1.6%)
Inositol phosphate metabolism 1 (0.8%)
Histidine metabolism 5 (4%)
Glyoxylate and dicarboxylate metabolism 6 (4.8%)
Glycolysis / Gluconeogenesis 1 (0.8%)
Glycine, serine and threonine metabolism 9 (7.2%)
Glycerophospholipid metabolism 6 (4.8%)
Glycerolipid metabolism 2 (1.6%)
Glutathione metabolism 3 (2.4%)
Galactose metabolism 2 (1.6%)
Fructose and mannose metabolism 2 (1.6%)
Folate biosynthesis 2 (1.6%)
Ether lipid metabolism 1 (0.8%)
Drug metabolism - other enzymes 1 (0.8%)
D-Glutamine and D-glutamate metabolism 1 (0.8%)
Cysteine and methionine metabolism 7 (5.6%)
Citrate cycle (TCA cycle) 4 (3.2%)
Carbon metabolism 7 (5.6%)
Caffeine metabolism 2 (1.6%)
C5-Branched dibasic acid metabolism 2 (1.6%)
Butanoate metabolism 2 (1.6%)
Biotin metabolism 3 (2.4%)
Biosynthesis of unsaturated fatty acids 2 (1.6%)
Biosynthesis of secondary metabolites - unclassified 3 (2.4%)
Biosynthesis of amino acids 15 (12%)
beta-Alanine metabolism 3 (2.4%)
Ascorbate and aldarate metabolism 4 (3.2%)
Arginine biosynthesis 3 (2.4%)
Arginine and proline metabolism 6 (4.8%)
Amino sugar and nucleotide sugar metabolism 3 (2.4%)
alpha-Linolenic acid metabolism 1 (0.8%)
Alanine, aspartate and glutamate metabolism 4 (3.2%)
2-Oxocarboxylic acid metabolism 8 (6.4%)
Metabolism
Vibrio cholerae infection 1 (0.8%)
Vancomycin resistance 2 (1.6%)
Prostate cancer 1 (0.8%)
Prion diseases 1 (0.8%)
Pertussis 2 (1.6%)
Pathways in cancer 2 (1.6%)
Parkinson disease 3 (2.4%)
Morphine addiction 1 (0.8%)
Human T-cell leukemia virus 1 infection 1 (0.8%)
Human papillomavirus infection 1 (0.8%)
Epithelial cell signaling in Helicobacter pylori infection 1 (0.8%)
Endocrine resistance 1 (0.8%)
Dilated cardiomyopathy (DCM) 1 (0.8%)
Cushing syndrome 2 (1.6%)
Cocaine addiction 2 (1.6%)
Choline metabolism in cancer 6 (4.8%)
Chemical carcinogenesis 1 (0.8%)
Chagas disease (American trypanosomiasis) 1 (0.8%)
Central carbon metabolism in cancer 9 (7.2%)
Breast cancer 1 (0.8%)
Antifolate resistance 2 (1.6%)
Amphetamine addiction 2 (1.6%)
Amoebiasis 1 (0.8%)
Alcoholism 2 (1.6%)
African trypanosomiasis 1 (0.8%)
Human Diseases
Sulfur relay system 1 (0.8%)
Aminoacyl-tRNA biosynthesis 7 (5.6%)
Genetic Information Processing
Sphingolipid signaling pathway 1 (0.8%)
Ras signaling pathway 1 (0.8%)
Rap1 signaling pathway 1 (0.8%)
Phospholipase D signaling pathway 1 (0.8%)
Phosphatidylinositol signaling system 1 (0.8%)
Neuroactive ligand-receptor interaction 1 (0.8%)
MAPK signaling pathway 1 (0.8%)
HIF-1 signaling pathway 2 (1.6%)
Hedgehog signaling pathway 1 (0.8%)
cGMP-PKG signaling pathway 2 (1.6%)
cAMP signaling pathway 3 (2.4%)
Calcium signaling pathway 1 (0.8%)
Apelin signaling pathway 1 (0.8%)
ABC transporters 11 (8.8%)
Environmental Information Processing
Oocyte meiosis 2 (1.6%)
Gap junction 2 (1.6%)
Ferroptosis 1 (0.8%)
Cellular Processes
0 6 12 20 28 36 44 52 60 68 76 84
Percent (%)

Figure S5. Metabolite KEGG classification map.

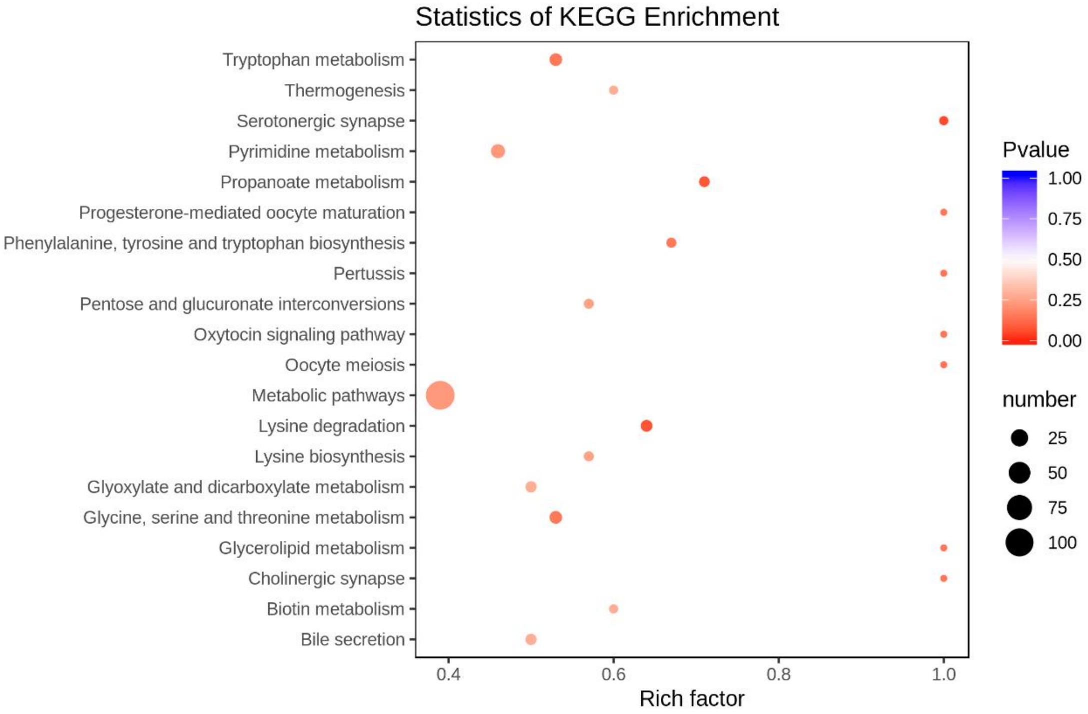

Figure S6. KEGG pathway enrichment scatter plot of the different metabolite.

Table S1 The gene related to digestion and metabolism

No.	Gene ID	Gene description
1	Cluster-18332.0	chlorophyll biosynthesis,
2	Cluster-3055.100960	cleavage of amino acids (exopeptidases) xaa-Pro aminopeptidase 1 [*Ostrinia furnacalis*]
3	Cluster-3055.101109	Actin binding protein
4	Cluster-3055.102325	energy metabolism AMP deaminase 2-like isoform X2 [*Spodoptera litura*]
5	Cluster-3055.102549	hydrolyses alpha bonds of large, alpha-linked polysaccharides
6	Cluster-3055.102639	enzyme regulator activity
7	Cluster-3055.102697	Catalytic enzyme
8	Cluster-3055.10788	threonine degradation to glycine
9	Cluster-3055.109444	Hydrolyzes a variety of dipeptides
10	Cluster-3055.110020	L-Kynurenine hydrolase Catabolism
11	Cluster-3055.112480	Detoxification
12	Cluster-3055.113116	breaking down complex carbohydrates
13	Cluster-3055.116424	involved in biosynthesis, intermediary metabolism and detoxification.
15	Cluster-3055.126242	enzyme involved in starch biosynthesis
16	Cluster-3055.131299	Mannosyltransferase involved in glycosylphosphatidylinositol-anchor biosynthesis

17	Cluster-3055.134879	melatonin biosynthesis
18	Cluster-3055.137587	diverged evolution, including digestive process
19	Cluster-3055.141280	Detoxification
20	Cluster-3055.141789	Detoxification
21	Cluster-3055.142283	digestive enzyme
22	Cluster-3055.147306	detoxification of xenobiotics acetylcholinesterase-like protein [*Glyphodes pyloalis*]
23	Cluster-3055.160929	digestive enzyme
24	Cluster-3055.25828	in stomach that converts ethanol to acetaldehyde
25	Cluster-3055.27418	detoxification cytochrome P450 6B2-like [*Ostrinia furnacalis*]
26	Cluster-3055.30387	reduction of glyoxylate to glycolate, digestive enzyme thioredoxin domain-containing protein 15 [*Ostrinia furnacalis*]
27	Cluster-3055.32110	involved in biosynthesis, intermediary metabolism and detoxification.
28	Cluster-3055.33090	hydrolyze sucrose aiming to produce inverted sugar
29	Cluster-3055.34069	digestive enzyme
30	Cluster-3055.64656	Antioxidants protein dj-1beta-like isoform X1 [*Ostrinia furnacalis*]

Table S2 Primers for the mutation genes in two species quantifications by qPCR

Primer name	Sequence (5'-3')
alpha-amylase-Sense	CAATGGACAACCAACAGA
alpha-amylase-Antisense	TCCCAGTTGTCGTTGTTGAA
CYP6AE76-Sense	GCCAAGTTCCCAGAGGAGCCAT
CYP6AE76-Antisense	GGAGTGAGGTTCTGCCGAAGC
RP49-Sense	GGCGTAAACCCAGAGGTATTGA
RP49-Antisense	TGTGACGGGTCTTCTTGTTTGAT

Table S3 Recombinant protein related information

Species	Protein	Molecular weight (kDa)	The name of the plasmid	Enzyme cutting site
C. punctiferalis	alpha-amylase	57.03	Pczn1	NdeI-XbaI
	CYP6AE76	59.23	Pczn1	NdeI-XbaI
C. pinicolalis	alpha-amylase	56.22	Pczn1	NdeI-XbaI
	CYP6AE76	59.71	Pczn1	NdeI-XbaI
